# Comparing the motivational underpinnings of sustainable consumption across contexts using a scenario-based approach

**DOI:** 10.3389/fpsyg.2022.854093

**Published:** 2022-09-23

**Authors:** Rouven Doran, Simen Bø, Daniel Hanss

**Affiliations:** ^1^Department of Psychosocial Science, University of Bergen, Bergen, Norway; ^2^Department of Strategy and Management, Norwegian School of Economics, Bergen, Norway; ^3^Department of Social Sciences, Darmstadt University of Applied Sciences, Darmstadt, Germany

**Keywords:** home, vacation, consumption motives, purchasing behavior, sustainable tourism

## Abstract

A sample of tourists (*N* = 780) responded to a survey addressing purchasing intentions and consumption motives in relation to buying sustainable groceries at a local food market. These intentions and motives were contrasted for two consumption contexts: on vacation vs. at home. An initial analysis of the data indicated that self-reported purchasing intentions were weaker for a vacation scenario than for a home scenario. Further analyses suggested that motives associated with purchasing intentions were not universal between contexts. At home, normative motives (i.e., good conscience) were positively associated with intentions, whereas other motives failed to explain significant variance (i.e., value for money, calm and safe, avoid boredom, pleasure, and good impression). On vacation, associations with intentions followed a similar pattern, except for the finding that hedonic motives (i.e., pleasure) added explanatory variance. Despite the increased importance of hedonic motives on vacation compared to at home, normative motives showed the strongest association with purchasing intentions in both consumption contexts. The findings are discussed with reference to the literature on contextual discrepancies in environmental behavior, while noting possible implications for promoting sustainable consumption among tourists.

## Introduction

The tourism sector is associated with a range of environmental and sociocultural changes, in addition to having impacts on the economy, especially in developing countries (Rutty et al., 2015). This becomes evident, for instance, when considering that the sector stands for a non-neglectable share of global greenhouse gas (GHG) emissions. More specifically, the volume of sectorial emissions has been approximated to 8% of the global GHG emissions, part of which can be attributed to activities resulting from and relating to individual consumption (Lenzen et al., 2018). While this makes these activities a potential target to keep emission growth within bounds, some scholars have argued that those who are worn out from mitigative efforts in their everyday life could be prone to rebound effects once they are on vacation (Hall et al., 2013). It is because of this that understanding the reasons for why individual consumers could hesitate to show the same level of engagement across contexts promises to yield important insights for policy strategies and behavioral interventions that seek to promote sustainable forms of tourism.

With respect to consumption decisions that take place after people have arrived at their destination, the reasons for diverting from everyday life can be as manifold as the motivations for which people choose to travel in the first place.^1^ Some consumers may choose the cheapest available option because it leaves room in their remaining travel budget, others may prefer products that provide a pleasant experience such as through the consumption of luxurious food, some may end up choosing a product based on the desire to escape the boredom of their regular shopping routines back home, and yet others may opt for an option that adheres to sustainable development goals. This study focuses on the latter and draws upon psychological literature that delineates motives that could drive decisions favoring products with sustainability attributes; see Steg and Nordlund (2019) for an overview of theories commonly used to explain environmental behavior.

## Literature review

The effort a person may invest to protect the environment in everyday life rarely accounts for their respective activities as tourists. Dolnicar and Grün (2009) surveyed a sample of tourists about their environmentally friendly behaviors across different contexts. Only a relatively small share of their sample showed the same behaviors on vacation as at home (e.g., purchases of refillable or reusable products, recycling of newspapers or cans). And related to this, the participants provided plenty of reasons (e.g., that they would spend most time at home). MacInnes et al. (2022) echoed these findings in the sense that a large share of their sample diverted from regular environmental engagement they normally displayed at home. Reasons that were provided by the participants for not displaying environmentally friendly behaviors on vacation varied in specific cases; for instance, experienced time pressure was mentioned as a reason for not eating leftovers but not in the context of avoiding heating. This complements some broader scholarly debate addressing contextual spill-over of environmentally friendly behaviors, including various aspects of sustainable consumption (Nash et al., 2017; Frezza et al., 2019).

Goal-framing theory (Lindenberg and Steg, 2007) asserts that the manner in which people select, process, and act upon available information depends on the relative strength of three overarching goals. These are described as hedonic goals (such as the perceived pleasantness of the target behavior), gain goals (such as the efficient use of disposable personal resources), and normative goals (such as the perceived appropriateness of the target behavior). While multiple goals may operate at the same time, it is the one that is most focal in the situation at hand that will exceed the greatest influence on behavioral decisions, whilst others may still remain influential in the background (Lindenberg and Steg, 2007). For instance, the physical presence of another person who picks sustainable groceries instead of conventional alternatives may underline the social acceptability of this behavior, which by extension would increase the relative impact of normative goals on individual purchasing decisions. In addition to individual value endorsements that could lead people to pay increased attention to certain behavioral alternatives, the focal strength of each goal can differ based on situational cues (Steg et al., 2014, 2016).

This theoretical perspective has been employed in several empirical studies seeking to understand individual differences in consumer behavior. Tang et al. (2020) investigated whether motives derived from the three overarching goals are associated with behaviors such as the purchasing of eco-labeled products, water conservation, and paper recycling. Their results showed that the anticipation of positive emotions (corresponding to the hedonic goal) and the desire to do what is deemed appropriate (corresponding to the normative goal) both explained variation in the propensity to engage in these behaviors. Cost-benefit calculations (corresponding to the gain goal) were unrelated to the behaviors. Thøgersen and Alfinito (2020) tested the effects of a goal framing manipulation on the importance of product attributes for a choice between organic and non-organic food. A normative goal frame increased the tendency to choose organic food, whereas a hedonic goal frame increased the importance of the physical appearance of the food on people’s preferences. These findings complement other literature that has employed a goal framing approach to understand individual consumption patterns (Chakraborty et al., 2017; Onel and Mukherjee, 2017; Shin and Kang, 2021; see also Barbopoulos and Johansson, 2016, for a related discussion).

When it comes to empirical studies that have explored the role of these motives for understanding tourism activities in particular, there has been a focus on hotel guests. Miao and Wei (2013) found that routine behaviors, such as switching off the lights, were shown less often in hotel rooms compared to what was regular at home. While normative considerations showed the strongest association with the investigated behaviors in a household context, the same behaviors in a hotel context were most strongly associated with hedonic considerations. Miao and Wei (2016) reported evidence to suggest that non-environmental aspects like time or effort can be linked to environmental behavior in hotels, again highlighting the role of hedonism in understanding tourism activities. Rodriguez–Sanchez et al. (2020) reported that hedonic motives (e.g., personal comfort) were negatively associated with water conservation during a hotel stay, whereas a positive association was reported for normative motives (e.g., felt obligation). And again, the association with conservation efforts turned out to be stronger for hedonic motives than for normative motives.

## Research aims

A contextual emphasis on relaxation and enjoyment has been suggested as an explanation for why people may show less environmental behavior during their vacation compared to if they are at home (Dolnicar et al., 2019). The reviewed literature supports this view insofar that an increased focus on personal comfort can be associated with reduced efforts to conserve resources in hotels. One issue that deserves further attention is whether these observations can be generalized toward the activities by tourists at large, for instance when they are shopping for groceries. If the assumption holds that the fulfillment of personal needs becomes especially salient on vacation, the situational focus on hedonic motives can have implications for a wider range of consumption-related activities.^2^ In the following, we report an empirical study that tests this claim, based on scenarios in which participants were asked to imagine buying groceries at a local food market. Several studies have pointed to the importance of food in shaping consumer experiences in tourism, especially when it is locally produced (Björk and Kauppinen-Räisänen, 2016; Birch and Memery, 2020).

## Materials and methods

### Participants

Participants were recruited among tourists who were visiting the city of Bergen (Norway) during the holiday (summer) season, *N* = 780, 18–91 years, *M*_*age*_ = 41.97, *SD*_*age*_ = 16.09. Data was collected at a vantage point near the city center, which provides a scenic overview of the city and the surrounding area. This site was chosen since it typically attracts visitors from a large variety of backgrounds, as well as with different modes of traveling. Research assistants approached potential respondents directly at the site, asking if they were currently on vacation. In the case of an affirmative response, the research assistants asked whether they would be willing to fill out a questionnaire addressing different facets of their experiences as tourists. Participation was voluntary, and without financial incentive. Female respondents (54.4%) were slightly more represented in comparison to male respondents (45.6%). For more details on the socio-demographic characteristics of the sample, including participants’ last night accommodation, see Table 1.

**TABLE 1:** Sample profile.

	*n*	%
**Gender**		
Female	424	54.4
Male	356	45.6
**Age**		
18–24	120	15.4
25–34	205	26.3
35–44	108	13.8
45–54	139	17.8
55–64	124	15.9
≥ 65	84	10.8
**Accommodation^a^**		
Camping facility	82	10.5
Private pension	39	5.0
HI hostel	17	2.2
Hotel	255	32.7
Cruise ship	161	20.6
Not specified	222	28.5
**Continent** ^b^		
Europe	546	70.0
North America	134	17.2
South America	18	2.3
Oceania	22	2.8
Asia	54	6.9
Africa	1	0.1
**Tourist type**		
International	743	95.3
Domestic	28	3.6

Percentages of responses within each category (separated by empty rows) do not always add up to 100 due to missing values (*n* = 4 for accommodation, *n* = 5 for continent, *n* = 9 for tourist type).

*^a^*Participants reported on their last night accommodation.

*^b^*Participants reported on their current place of residence.

### Procedures

Each participant filled out a paper-and-pen questionnaire. To enable us to test whether the motivational underpinning of intentions to buy sustainable groceries differs between contexts, participants filled out the measures of the target variables after having been asked to imagine visiting a local food market in a home scenario vs. a vacation scenario. Using imagined scenarios compared to standard questions helps to make the situations more concrete and approximate of real-life situations (Alexander and Becker, 1978). While the study assesses cross-sectional associations between consumption motives and purchasing intentions, a repeated measures design was employed to examine possible differences in associative patterns across consumption contexts. The sequence in which items pertaining to either one of the two scenarios were presented in the questionnaire was counterbalanced between participants to account for possible order effects. An overview of means and standard deviations for each context scenario can be found in Table 2.

**TABLE 2:** Means and standard deviations for item measures.

	Home			Vacation		
	*n*	*M*	*SD*	*n*	*M*	*SD*
**Purchasing intentions (1 = Very unlikely, 7 = Very likely)**						
When I buy wrapped food, I will make sure that the wrapping can be recycled.	749	3.77	1.21	754	3.33	1.26
When I buy fruits and vegetables and have the choice between ecological and conventional products, I will buy ecological products.	746	3.87	1.10	755	3.48	1.19
When I buy food and have the choice, I will buy products that guarantee fair payment to the producers.	748	3.92	1.03	755	3.59	1.15
When I buy fruits and vegetables, I will go to a farmers’ market or a similar place where I can buy directly from the farmer.	749	3.35	1.31	758	2.99	1.30
**Consumption motives (1 = Not at all important, 7 = Extremely important)**						
Value for money: I should get a lot for the price I pay^a^	744	5.34	1.23	755	4.70	1.29
Fulfills expectations: the product should fulfill even my highest requirements and expectations^a^	741	5.53	1.07	749	5.21	1.10
Calm and safe: the product should make me feel calm and safe^a^	732	5.24	1.46	744	5.18	1.50
Avoid boredom: It is important that the product is not too boring or routine^a^	732	4.60	1.54	739	5.20	1.54
Pleasure: the product should be pleasant and agreeable^a^	727	5.49	1.14	738	5.65	1.10
Good conscience: the product should give me a good conscience^a^	732	5.51	1.25	743	5.29	1.31
Good impression: the product should make a good impression on people who are important to me^a^	736	4.55	1.82	745	4.57	1.86

This table summarizes means and standard deviations for items measuring purchasing intentions and consumption motives. Note that for consumption motives, the questionnaire displayed the first words of each item (e.g., “Value for money”) in boldface. The sequence in which items regarding each consumption context were presented in the questionnaire was counterbalanced; for more details, see “Materials and methods” section. ^a^Adopted from Barbopoulos and Johansson, 2017a,b.

### Measures

Consumption motives were measured with seven items adopted from the Consumer Motivation Scale (CMS; Barbopoulos and Johansson, 2017a,b). Each item captured a unique motive and asked respondents how important this motive was to them personally (answer scale: 1 = Not at all important, 7 = Extremely important). The seven motives each related to one of three goals: The gain goal (motives: value for money, fulfills expectations, calm and safe), the hedonic goal (motives: avoid boredom, pleasure), and the normative goal (motives: good conscience, good impression). Items were introduced as follows: “Imagine that you are visiting a local food market that offers a wide range of products. These products differ in many ways including their environmental and social impacts etc. How important are the following aspects … [… when you are at home? … when you are on vacation?]”.

Purchasing intentions were assessed with four items, each concerned with the likelihood to which participants would purchase grocery products with different sustainability attributes (answer scale: 1 = Very unlikely, 5 = Very likely). This selection of attributes (recyclable, fair trade, ecological, and bought directly from the farmer) was informed by prior research indicating that environmental and social aspects are common in consumers’ concept of sustainable groceries (Hanss and Böhm, 2012). Items were introduced as follows: “Imagine the same situation as described above, namely that you are visiting a local food market that offers a wide range of products. How likely is it that you make the following choices … [… when you are at home? … when you are on vacation?]”. Purchasing intentions were analyzed with composite scores that reflected average scores across the four items, computed separately for the home scenario (α = 0.77, *M* = 3.72, *SD* = 0.91) and the vacation scenario (α = 0.80, *M* = 3.34, *SD* = 0.97).

## Results

**FIGURE 1:**
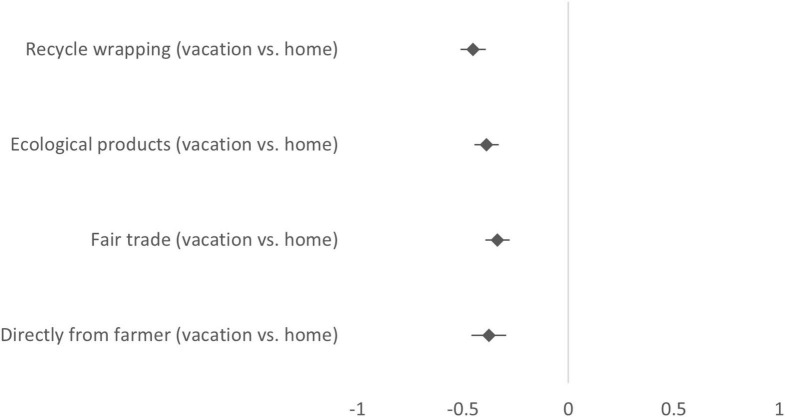
Purchasing intentions when visiting a local food market, compared between contexts (answer scale: 1 = Very unlikely, 5 = Very likely). Mean differences (diamonds) are shown with 95% confidence intervals (bars). A positive value indicates that the likelihood to purchase sustainable products was rated higher in the vacation scenario than in the home scenario, a negative value indicates that it was rated lower.

Figure 1 shows mean differences in self-reported purchasing intentions, plotted separately for each sustainability attribute. A dependent *t*-test based on the composite scores of purchasing intentions yielded significant mean differences. More precisely, participants were on average more likely to intend purchasing sustainable groceries at a food market when being at home than when being on vacation [*t*(746) = 16.21, *p* < 0.001, *d* = 0.41].

**FIGURE 2:**
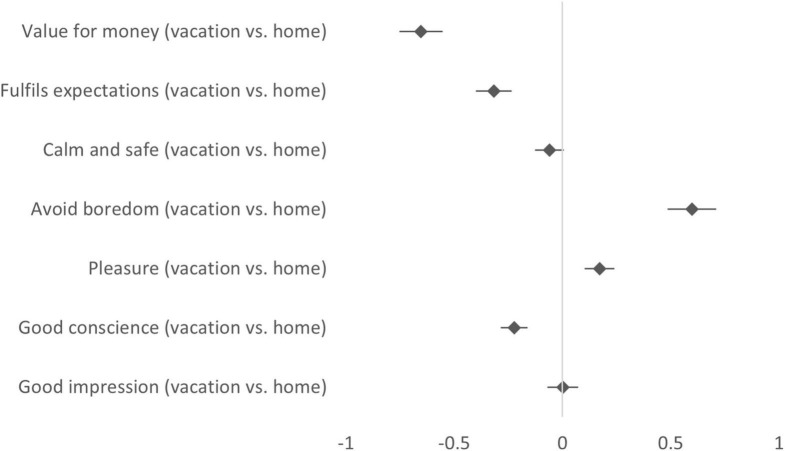
Consumption motives when visiting a local food market, compared between contexts (answer scale: 1 = Not at all important, 7 = Extremely important). Mean differences (diamonds) are shown with 95% confidence intervals (bars). A positive value indicates that the importance of the motive was rated stronger in the vacation scenario than in the home scenario, a negative value indicates that it was rated weaker.

Figure 2 depicts mean differences in self-reported consumption motives, shown separately for each motive category. A series of dependent *t*-tests (Bonferroni corrected) indicated that perceived importance of specific consumption motives differed between the imagined scenarios. In connection with the vacation scenario, participants were less likely to emphasize gain motives such as value for money [*t*(735) = –12.89, *p* < 0.001, *d* = –0.52] and that the purchase would fulfill expectations [*t*(730) = –7.61, *p* < 0.001, *d* = –0.29]. They also put less emphasis on normative motives in the form of having a good conscience [*t*(724) = –7.14, *p* < 0.001, *d* = –0.17]. Participants were in the meantime more likely to consider hedonic motives such as avoiding boredom [*t*(720) = 10.44, *p* < 0.001, *d* = 0.39] and seeking pleasure [*t*(717) = 4.84, *p* < 0.001, *d* = 0.15], when they considered consumption at a local food market on vacation compared to at home. Two additional motives that were labeled as calm and safe [*t*(722) = –1.80, *p* = 0.073, *d* = –0.04] and good impression [*t*(725) = 0.04, *p* = 0.970, *d* = 0.00] did not differ in their perceived importance between the context scenarios.

Table 3 juxtaposes results from two multiple regression analyses to investigate the predictive value of different consumption motives for choosing sustainable groceries on vacation vs. at home.^3^ Results indicate that having a good conscience (as a normative motive) was significantly and positively associated with intentions to make sustainable purchases, both at home and on vacation. However, pleasure (as a hedonic motive) showed differential associations. It was significantly and negatively associated with intentions to buy sustainable groceries on vacation, but not significantly associated with purchasing intentions in the home scenario. The remaining consumption motives failed to show any significant associations with purchasing intentions, irrespective of whether consumption decisions were imagined as taking place at home or on vacation.

**TABLE 3:** Multiple regressions predicting purchasing intentions from consumption motives.

	Home			Vacation		
	*B*	95% CI	β	*B*	95% CI	β
Constant	2.61	[2.17, 3.05]		2.09	[1.65, 2.54]	
**Gain motives**						
Value for money	–0.04	[–0.10, 0.02]	–0.05	0.02	[–0.03, 0.08]	0.03
Fulfills expectations	0.05	[–0.02, 0.13]	0.06	0.04	[–0.04, 0.11]	0.04
Calm and safe	0.02	[–0.03, 0.07]	0.03	0.03	[–0.02, 0.08]	0.04
**Hedonic motives**						
Avoid boredom	0.01	[–0.04, 0.06]	0.01	–0.01	[–0.06, 0.04]	–0.01
Pleasure	–0.05	[–0.12, 0.02]	–0.06	–0.09	[–0.17, –0.02]	–0.11*
**Normative motives**						
Good conscience	0.21	[0.15, 0.27]	0.29***	0.22	[0.16, 0.28]	0.30***
Good impression	0.00	[–0.04, 0.04]	–0.01	0.04	[0.00, 0.08]	0.07
*R* ^2^	0.09			0.11		
*R* ^2^_Adjusted_	0.08			0.11		
*F*	(7, 686) = 9.46***			(7, 702) = 12.94***		

Listwise deletion. *N* = 694 for home scenario, *N* = 710 for vacation scenario. CI, confidence interval for unstandardized regression coefficients (B). *p < 0.05 and ***p < 0.001.

## Discussion

Tourism comprises the “activities of persons traveling to and staying in places outside their usual environment for not more than one consecutive year for leisure, business and other purposes” (UNWTO, 1995, p. 1). Many images in popular culture portray these activities in connection with beach resorts, yet these descriptions fall short in capturing the complexity of tourism as a socio-cultural phenomenon. Some individuals may decide to go on vacation primarily because they look for comfort and relaxation, others could book a weekend trip to a foreign city in the anticipation of experiencing cultures that are different from their own, and yet others may be driven by the desire to reunite with family and friends during the holiday season. These examples illustrate that the motives that drive people to travel away from home, and by extension the type of experiences that they seek from related consumption decisions, can be quite heterogenous (see Heitmann, 2011; Dann, 2018).

These activities—despite their heterogeneity—can provide a means by which people can break out from the boundaries that are imposed on them in everyday life. This becomes reflected in the desire for an interim escape from regular routines and responsibilities, along with the wish to encounter positive experiences whilst being at the destination, both of which are traditionally viewed as core motivations for why people travel away from home (Dann, 1977; Iso-Ahola, 1982). The reported analyses support this view by showing that motives for boredom avoidance and pleasure seeking were more pronounced in the vacation scenario than in the home scenario, accompanied with a reduced emphasis on motives like having a good conscience when imagining a local food market visit as tourists. Taken together, these results support the notion that tourism constitutes a context where the subjective importance assigned to specific motives tends to differ from everyday life.

It is a core premise in goal-framing theory that cues supporting hedonic and/or gain goals can push normative goals into the cognitive background, which for the latter implies less influence on behavioral decisions (Lindenberg and Steg, 2007). Drawing upon this theoretical insight, the current study explored if an assumed situational focus on hedonism can account for some of the contextual variation in consumption-related activities. The specific assumption was that when a person operates within their regular context (at home) and the pursuit of pleasure gets relatively less emphasized, features associated with shopping in public, like the physical presence of other consumers, can resonate well with normative considerations regarding the need to preserve the environment. If the same person acts outside this context (on vacation) and the seeking of aggregable experiences becomes increasingly important, however, the relevance of normative considerations might be lessened. Despite evidence that the individual motivation for (not) behaving environmentally friendly may indeed differ from context to context (Miao and Wei, 2013), it is only recently that scholars have employed this perspective to understand food consumption during vacation (Liu et al., 2022).

There was one consumption motive that showed a negative significant association with product purchases when participants were asked to imagine visiting a local food market on vacation: the more participants regarded the pleasantness and agreeableness of a product as important, the less likely were they to choose sustainable groceries in this scenario. However, and contrary to our initial assumption, the predictive value of this hedonic motive continued to be comparatively lower than that for the normative motive of having a good conscience. The latter was also the only consumption motive showing a consistent significant association with purchasing intentions, regardless of where these decisions were imagined taking place. While the results support the view that a situational focus on personal comfort may constrain environmentally friendly behavior, they do not corroborate prior studies in which this particular motive outperformed normative considerations as a predictor for conservation efforts among hotel guests (Miao and Wei, 2016; Rodriguez–Sanchez et al., 2020).

In sum, the current findings would seem to suggest that signaling the importance of behaving responsibly as a consumer could be a strategy to motivate purchases of products with sustainability attributes across contexts. This can be done, for instance, by means of persuasive messages that express gratitude toward grocery shoppers for doing their part in supporting the environment (e.g., reusing their plastic bags; De Groot et al., 2013). If the context is such that hedonic motives increase in their importance, as it has been demonstrated in the scenario that involved making product choices on vacation, accentuating the personal gains resulting from these choices may be of additional benefit. Along appeals toward a person’s sense of responsibility to take action that serves society at large, campaigners could stress areas where lower environmental impacts potentially enhance the quality of one’s own tourist experience. An example could be to market certain qualities of the product itself, such as for instance the opportunity to experience the unique taste from locally produced food at the destination. This interpretation follows the idea that, in addition to strengthening the salience of normative goals, resolving conflicts with hedonic or gain goals can provide an alternative route to promote environmental behavior (Steg et al., 2014, 2016).

Since the identified motives explained only a small fraction of the variance in each regression model, there is room to speculate about other factors that may determine individual differences in dietary habits and food preferences (for an overview and discussion, see Vermeir et al., 2020). Including a broader selection of possible determinants would yield a more robust assessment of the comparative importance of hedonic and normative motives, both within and across different consumption contexts. It might be particularly worth looking at the role of non-psychological aspects, such as the availability or price of the product itself. This should ideally be supplemented with measures on perceived barriers, which, in combination to the intentions assessed in this study, may help to better predict sustainable purchases. The available literature has identified a variety of reasons for which consumers may not act on their intentions to buy local food, for instance, including but not limited to a lack of trust (Birch and Memery, 2020).

## Limitations

The following issues remain to be addressed by future studies. First, each participant was approached at the same destination, which may have introduced a sampling bias as specific destinations may be particularly appealing to a certain visitor profile. Replications that comprise visitors at other destinations are warranted to test if the identified associations remain robust. Second, the questionnaire asked participants to imagine the scenario of visiting a local food market at home vs. on vacation. While there are cases in which mental simulation can substitute for actual experience in terms of having similar cognitive and behavioral effects (Kappes and Morewedge, 2016), the presented scenario may have less experimental realism than measuring behavior while participants are physically at each respective location (Morales et al., 2017). Third, the data was collected prior to the outbreak of the COVID-19 pandemic, which has disrupted consumption patterns and tourism activities in many countries. Some research indicates, for instance, that there has been an increased emphasis on utilitarian motivations in consumer decisions in response to the crisis (Vázquez-Martínez et al., 2021). At this point, it remains unclear how different motives for consumption may have changed with regards to tourism activities that take place during the pandemic, and if that should be the case, to what extent these changes continue to persist.

## Conclusion

It is known that people employ various justifications to explain their reduced environmental engagement when they are away from home, such as for instance by emphasizing that holidays are an exception (Juvan and Dolnicar, 2014; Juvan et al., 2016). Rather than focusing on how people attempt to make sense of their behavior in retrospect, the present study compared intentions to purchase sustainable groceries at a local food market, followed by an empirical analysis of their motivational underpinning. People were more likely to plan on purchasing sustainable groceries when they imagined visiting a local food market at home, than when they imagined making these choices on vacation. Whereas purchasing intentions were in general more strongly associated with having a good conscience than with pleasure seeking, the explanatory value of the latter remained limited toward the vacation scenario. These findings suggest that social marketers may want to consider tailoring the content of their campaigns toward the specific consumption context at hand, and where applicable, address how choosing products with low environmental impacts may still result in pleasant experiences.

## Data availability statement

The raw data supporting the conclusions of this article will be made available by the authors, without undue reservation, to any qualified researcher.

## Ethics statement

Ethical review and approval was not required for the study on human participants in accordance with the local legislation and institutional requirements. Informed consent was inferred by participants filling out the questionnaire.

## Author contributions

RD and DH contributed to the conception and design of the study. RD organized the data collection and wrote the first draft of the manuscript. RD and SB performed the statistical analysis. SB and DH wrote sections of the manuscript. All authors contributed to manuscript revision, read, and approved the submitted version.

## Footnotes

1 For a further discussion on the literature on tourist motivation, see Heitmann (2011) and Dann (2018).

2 While one can think of several examples in which environmental engagement may infringe upon personal needs, the focus on pleasure seeking can make these tensions even more salient when people are on vacation (Dolnicar et al., 2008).

3 To further scrutinize the identified patterns of associations, we reran the statistical analyses with participants’ last night accommodation as a covariate, using contrast coding with hotels as the baseline. Results were remarkably similar to those reported here. For reasons of parsimony, this paper only reports on the analyses that exclusively incorporated measures addressing different consumption motives to explain individual differences in purchasing intentions.

## References

[B1] Alexander C. S. Becker H. J. (1978). The use of vignettes in survey research. *Public Opin. Q.* 42:93. 10.1086/268432

[B2] Barbopoulos I. Johansson L.-O. (2016). A multi-dimensional approach to consumer motivation: Exploring economic, hedonic, and normative consumption goals. *J. Consum. Mark.* 33 75–84. 10.1108/JCM-08-2014-1091

[B3] Barbopoulos I. Johansson L.-O. (2017a). The Consumer Motivation Scale: Development of a multi-dimensional and context-sensitive measure of consumption goals. *J. Bus. Res.* 76 118–126. 10.1016/j.jbusres.2017.03.012

[B4] Barbopoulos I. Johansson L.-O. (2017b). The Consumer Motivation Scale: A detailed review of item generation, exploration, confirmation, and validation procedures. *Data Brief* 13 88–107. 10.1016/j.dib.2017.04.054 28580406 PMC5447383

[B5] Birch D. Memery J. (2020). Tourists, local food and the intention-behaviour gap. *J. Hosp. Tour. Manag.* 43 53–61. 10.1016/j.jhtm.2020.02.006

[B6] Björk P. Kauppinen-Räisänen H. (2016). Local food: A source for destination attraction. *Int. J. Contemp. Hosp. Manag.* 28 177–194. 10.1108/IJCHM-05-2014-0214

[B7] Chakraborty A. Singh M. P. Roy M. (2017). A study of goal frames shaping pro-environmental behaviour in university students. *Int. J. Sustain. High. Educ.* 18 1291–1310. 10.1108/IJSHE-10-2016-0185

[B8] Dann G. M. S. (1977). Anomie, ego-enhancement and tourism. *Ann. Tour. Res.* 4 184–194. 10.1016/0160-7383(77)90037-8

[B9] Dann G. M. S. (2018). “Why, oh why, oh why, do people travel abroad?,” in *Creating Experience Value in Tourism*, 2nd Edn, eds Prebensen N. K. Chen J. S. Uysal M. (Wallingford: CABI), 44–56. 10.1079/9781786395030.0044

[B10] De Groot J. I. M. Abrahamse W. Jones K. (2013). Persuasive normative messages: The influence of injunctive and personal norms on using free plastic bags. *Sustainability* 5 1829–1844. 10.3390/su5051829

[B11] Dolnicar S. Crouch G. I. Long P. (2008). Environment-friendly tourists: What do we really know about them? *J. Sustain. Tour.* 16 197–210. 10.2167/jost738.0

[B12] Dolnicar S. Grün B. (2009). Environmentally friendly behavior: Can heterogeneity among individuals and contexts/environments be harvested for improved sustainable management? *Environ. Behav.* 41 693–714. 10.1177/0013916508319448

[B13] Dolnicar S. Knezevic Cvelbar L. Grün B. (2019). A sharing-based approach to enticing tourists to behave more environmentally friendly. *J. Travel Res.* 58 241–252. 10.1177/0047287517746013 30662098 PMC6318704

[B14] Frezza M. Whitmarsh L. Schäfer M. Schrader U. (2019). Spillover effects of sustainable consumption: Combining identity process theory and theories of practice. *Sustainability* 15 15–30. 10.1080/15487733.2019.1567215

[B15] Hall C. M. Scott D. Gössling S. (2013). The primacy of climate change for sustainable international tourism. *Sustain. Dev.* 21 112–121. 10.1002/sd.1562

[B16] Hanss D. Böhm G. (2012). Sustainability seen from the perspective of consumers. *Int. J. Consum. Stud.* 36 678–687. 10.1111/j.1470-6431.2011.01045.x

[B17] Heitmann S. (2011). “Tourist behaviour and tourism motivation,” in *Research Themes for Tourism*, eds Robinson P. Heitmann S. Dieke P. (Wallingford: CABI), 31–44. 10.1079/9781845936846.0031

[B18] Iso-Ahola S. E. (1982). Toward a social psychological theory of tourism motivation: A rejoinder. *Ann. Tour. Res.* 9 256–262. 10.1016/0160-7383(82)90049-4

[B19] Juvan E. Dolnicar S. (2014). The attitude–behaviour gap in sustainable tourism. *Ann. Tour. Res.* 48 76–95. 10.1016/j.annals.2014.05.012

[B20] Juvan E. Ring A. Leisch F. Dolnicar S. (2016). Tourist segments’ justifications for behaving in an environmentally unsustainable way. *J. Sustain. Tour.* 24 1506–1522. 10.1080/09669582.2015.1136635

[B21] Kappes H. B. Morewedge C. K. (2016). Mental simulation as substitute for experience. *Soc. Pers. Psychol. Compass* 10 405–420. 10.1111/spc3.12257

[B22] Lenzen M. Sun Y.-Y. Faturay F. Ting Y.-P. Geschke A. Malik A. (2018). The carbon footprint of global tourism. *Nat. Climate Change* 8 522–528. 10.1038/s41558-018-0141-x

[B23] Lindenberg S. Steg L. (2007). Normative, gain and hedonic goal frames guiding environmental behavior. *J. Soc. Issues* 63 117–137. 10.1111/j.1540-4560.2007.00499.x

[B24] Liu T. Juvan E. Qiu H. Dolnicar S. (2022). Context- and culture-dependent behaviors for the greater good: A comparative analysis of plate waste generation. *J. Sustain. Tour.* 30 1200–1218. 10.1080/09669582.2021.1918132

[B25] MacInnes S. Grün B. Dolnicar S. (2022). Habit drives sustainable tourist behaviour. *Ann. Tour. Res.* 92:103329. 10.1016/j.annals.2021.103329 PMC8592521 34803196

[B26] Miao L. Wei W. (2013). Consumers’ pro-environmental behavior and the underlying motivations: A comparison between household and hotel settings. *Int. J. Hosp. Manag.* 32 102–112. 10.1016/j.ijhm.2012.04.008

[B27] Miao L. Wei W. (2016). Consumers’ pro-environmental behavior and its determinants in the lodging segment. *J. Hosp. Tour. Res.* 40 319–338. 10.1177/1096348013495699

[B28] Morales A. C. Amir O. Lee L. (2017). Keeping it real in experimental research—Understanding when, where, and how to enhance realism and measure consumer behavior. *J. Consum. Res.* 44 465–476. 10.1093/jcr/ucx048

[B29] Nash N. Whitmarsh L. Capstick S. Hargreaves T. Poortinga W. Thomas G. (2017). Climate-relevant behavioral spillover and the potential contribution of social practice theory. *Wiley Interdiscip. Rev.* 8:e481. 10.1002/wcc.481

[B30] Onel N. Mukherjee A. (2017). Why do consumers recycle? A holistic perspective encompassing moral considerations, affective responses, and self-interest motives. *Psychol. Mark.* 34 956–971. 10.1002/mar.21035

[B31] Rodriguez–Sanchez C. Sancho-Esper F. Casado-Díaz A. B. Sellers-Rubio R. (2020). Understanding in-room water conservation behavior: The role of personal normative motives and hedonic motives in a mass tourism destination. *J. Destination Mark. Manag.* 18:100496. 10.1016/j.jdmm.2020.100496

[B32] Rutty M. Gössling S. Scott D. Hall C. M. (2015). “The global effects and impacts of tourism: An overview,” in *The Routledge Handbook of Tourism and Sustainability*, eds Hall C. M. Gossling S. Scott D. (Milton Park: Routledge), 10.4324/9780203072332

[B33] Shin H. W. Kang J. (2021). What motivates your environmentally sustainable stay? Exploration of the underlying mechanism of consumers’ intentions to use green peer-to-peer accommodations. *J. Travel Tour. Mark.* 38 413–430. 10.1080/10548408.2021.1921672

[B34] Steg L. Bolderdijk J. W. Keizer K. Perlaviciute G. (2014). An integrated framework for encouraging pro-environmental behaviour: The role of values, situational factors and goals. *J. Environ. Psychol.* 38 104–115. 10.1016/j.jenvp.2014.01.002

[B35] Steg L. Lindenberg S. Keizer K. (2016). Intrinsic motivation, norms and environmental behaviour: The dynamics of overarching goals. *Int. Rev. Environ. Resour. Econ.* 9 179–207. 10.1561/101.00000077

[B36] Steg L. Nordlund A. (2019). “Theories to explain environmental behaviour,” in *Environmental Psychology: An Introduction*, 2nd Edn, eds Steg L. de Groot J. I. M. (Hoboken, NJ: John Wiley & Sons Ltd), 217–227.

[B37] Tang Y. Chen S. Yuan Z. (2020). The effects of hedonic, gain, and normative motives on sustainable consumption: Multiple mediating evidence from China. *Sustain. Dev.* 28 741–750. 10.1002/sd.2024

[B38] Thøgersen J. Alfinito S. (2020). Goal activation for sustainable consumer choices: A comparative study of Denmark and Brazil. *J. Consum. Behav.* 19 556–569. 10.1002/cb.1824

[B39] UNWTO (1995). *Technical Manual no. 2: Collection of Tourism Expenditure Statistics.* Madrid: UNWTO.

[B40] Vázquez-Martínez U. J. Morales-Mediano J. Leal-Rodríguez A. L. (2021). The impact of the COVID-19 crisis on consumer purchasing motivation and behavior. *Eur. Res. Manag. Bus. Econ.* 27:100166. 10.1016/j.iedeen.2021.100166

[B41] Vermeir I. Weijters B. De Houwer J. Geuens M. Slabbinck H. Spruyt A. (2020). Environmentally sustainable food consumption: A review and research agenda from a goal-directed perspective. *Front. Psychol.* 11:1603. 10.3389/fpsyg.2020.01603 32754095 PMC7381298

